# Defining optimal orthogeriatric hip fracture care: a delphi consensus approach

**DOI:** 10.1007/s41999-025-01156-5

**Published:** 2025-02-06

**Authors:** H. E. van Bremen, L. J. Seppala, E. A. Gans, J. H. Hegeman, N. van der Velde, H. C. Willems

**Affiliations:** 1 Amsterdam Bone Center, Movement Sciences Amsterdam, Meibergdreef 9, 1105 AZ Amsterdam, The Netherlands; 2 Dutch Institute for Clinical Auditing, Leiden, The Netherlands; 3 Internal Medicine and Geriatrics, Amsterdam UMC Location University of Amsterdam, Amsterdam, The Netherlands; 4 Amsterdam Public Health Research Institute, Amsterdam, The Netherlands; 5 University Center of Geriatric Medicine, University Medical Center Groningen, Groningen, The Netherlands; 6 Knowledge Institute of the Dutch Association of Medical Specialists, Utrecht, The Netherlands; 7 Biomedical Signals and Systems Group, University of Twente, Enschede, The Netherlands; 8 Department of Trauma Surgery, Ziekenhuisgroep Twente, Almelo-Hengelo, The Netherlands

**Keywords:** Hip fracture, Delphi, Orthogeriatic care, Optimal care

## Abstract

**Purpose:**

Development of consensus-based recommendations on core and optimal elements of orthogeriatric hip-fracture care.

**Methods:**

An online Delphi survey was performed in the Netherlands. A total of 72 statements were derived from a framework encompassing all phases of care for older patients with a hip fracture. These statements were presented to the panelists in two rounds to identify elements for minimal and optimal orthogeriatric care. Panelists included professionals with experience in hip-fracture care and patient representatives. The level of agreement was measured using a 5-point Likert scale. Consensus was considered if > 75% of the panelists agreed or disagreed.

**Results:**

Ninety-two persons were invited to participate in the survey; 63 participated in the first round and 55 in the second round. One statement was added in the second round. Most participants had a background in geriatrics (36% in the second round) or trauma surgery/orthopaedics (20% in the second round). Consensus was reached on 48 statements for minimal orthogeriatric care and 60 statements for optimal orthogeriatric care.

**Conclusion:**

This study supports previously established recommendations for older adults with hip fractures. In addition, it offers practical recommendations for implementation of orthogeriatric care regarding both core and optimal care elements for hospitals at every different level of maturity and at every step in the care process. This may decrease the intra- and inter-hospital variability of clinical management of hip-fracture patients. Organizational and logistical elements present a barrier to overcoming the gap between the current practice and the optimal situation.

## Key summary points

**Aim:**

Defining core and optimal elements for hospitals providing orthogeriatric hip-fracture care at different maturity levels by using a consensus-based Delphi approach.

**Findings:**

48 core and 60 optimal elements were identified for orthogeriatric care.

**Message:**

The identified core and optimal elements offer practical recommendations for implementation of orthogeriatric care. Still, organizational and logistical elements present a barrier to bridging the gap between the current practice and optimal situation.

## Introduction

More than half of patients suffering a hip fracture are living with frailty [1]. Several characteristics of this population underline the frailty of the patients, including the high rate of comorbidity, reaching up to 99.8% [2]. It is known that hip-fracture patients have a high risk of perioperative medical and surgical complications, with rates reaching up to 50%, together with a notable postoperative early mortality rate [3–5].

Managing frailty requires expertise outside the surgical field. This expertise can be ensured through the implementation of orthogeriatric care. Orthogeriatric care is a medical-surgical model combining multidisciplinary health professionals from trauma surgery, orthopaedics, and geriatric medicine to treat older patients with a hip fracture holistically [6]. Orthogeriatric care has been proven effective in hip-fracture care, lowering the number of consultations, complications, and readmissions and increasing bone health assessment and mobility [7–10].

Previous literature on orthogeriatric care and related organizational documents have already laid solid groundwork for implementation of orthogeriatric care [6, 11, 12]. The Fragility Fracture Network (FFN) offers guidance for developing and optimizing care, including current evidence around integrated orthogeriatric management. This includes an orthogeriatric framework comprising various essential care components, along with a clinical toolkit and a special interest group for members of hip-fracture audits [13–15]. In addition, national guidelines are available, and hip-fracture registries, using several clinical indicators, have been developed to assess the quality of care for hip-fracture patients [16]. However, integrating current guidelines into everyday clinical practice and aligning them with local health structures can be challenging. Practical recommendations explicitly tailored to hospitals at various stages of implementing orthogeriatric care—ranging from those just beginning the process to those seeking to enhance current practices—are currently lacking.

The aim of this study was to further develop -consensus-based recommendations regarding elements of care that contribute to both minimal and optimal orthogeriatric care. This study partly builds upon previously established recommendations for patients with hip fractures, as outlined in existing literature and guidelines, including those from the FFN. In addition, it offers insights by introducing novel perspectives, such as insights in Nonoperative Management (NOM) and provides practical recommendations from the expert and patient perspective.

## Methods

### Study design

An online Delphi survey facilitated by Castor was conducted in the Netherlands following the recommendations for conducting and reporting Delphi studies (CREDES) [17]. The Delphi method is an established consensus method to assess the extent of agreement on a particular issue, in this case, ‘What are the (i) core and (ii) optimal orthogeriatric elements for delivering care to older patients with a hip fracture?’. The core elements collectively defined the minimal care standard, while the optimal elements represented the ideal orthogeriatric care. The Delphi method allowed us to synthesize diverse expert opinions while encouraging consensus-building and minimizing potential biases associated with group interactions. Panelists could express their opinions without the influence of the rest of the panel, as the Delphi rounds were undertaken remotely and anonymously. The Medical Ethics Research Committee of Amsterdam UMC declared that the Medical Research Involving Human Subjects Act did not apply to this study. Panel members provided written informed consent at the beginning of each questionnaire.

### Participant selection

All members from a specified project group for integration of orthogeriatric care set out to recruit a multidisciplinary panel through their networks. The panel included physicians or nurse practitioners working in anesthesiology, emergency care, trauma surgery, orthopedic surgery, geriatrics, internal medicine, or elderly care medicine, and allied health professionals (dietician, physiotherapist, and occupational therapist). Panelists needed to have experience in hip-fracture care in older adults and be from different regions of the Netherlands, working in academic and non-academic hospitals. In addition, patients and adults with relatives who suffered a hip fracture were recruited via a patient organization; two of them sustained a hip fracture in the past, and four had relatives who suffered a hip fracture.

### A framework of care components

To inform the first Delphi round, a model of relevant care elements was developed based on the national Dutch guidelines for hip-fracture care. [11, 12, 18] These care items were then refined and validated by the members of the designated project group, which included experts from various disciplines. The finalized items were integrated into a comprehensive framework. The framework followed the patient journey in the hospital (emergency care, preoperative care, postoperative care, discharge, and outpatient care). In addition, two topics were added to the framework: Shared Decision Making (SDM) and NOM. The initial set of statements was derived by one researcher (H.B.) from the guideline-based framework. Subsequently, L.J. and H.W. reviewed, evaluated, and refined the statements where necessary. Following this process, the finalized statements were subjected to a pilot phase to ensure clarity and relevance. A detailed description of the framework is available in the Appendix.

### Pilot

The survey was piloted among six healthcare professionals from surgical and geriatric specialisms to check the clarity and spelling of the questions and provide suggestions and comments on optimizing the questions.

### Delphi round 1

The invitation for the first round was sent in October 2023. The panelists were emailed a link to the internet-based questionnaire and given two weeks to complete it. Two reminders were sent if they had not responded. The panelists were asked to provide personal information, such as age, profession, years of experience in hip-fracture care, and whether they worked at a university-affiliated hospital. This was used to tailor the specific statements based on their profession. The Delphi panelists were asked to indicate to what extent they agreed to statements for the “minimal care” and “optimal care” for a hip-fracture patient. (Full overview of statements can be found in Appendix) Responses were measured using a Likert scale (1 = strongly agree, 2 = agree, 3 = neutral, 4 = disagree, 5 = strongly disagree). In addition, the option 'Outside the scope of my expertise' was added. An opportunity to comment on the items was given in the form of a free text field. Furthermore, the panelists were asked to propose missing care components and to explain why these should be included, preferably including a reference providing evidence supporting their argument. Specifically, patients were offered the opportunity for additional clarification via telephone regarding the care components. The facilitators (HB, HW, and LS) evaluated the responses. The percentages of (dis-)agreements were assessed.

### Delphi round 2

The invitation for the second round was sent in December 2023 to all panelists who participated in the first round. In the second round, items were presented for which no consensus had been reached by panelists in the first round. In addition, panelists were provided with the percentage of (dis-) agreement of round 1 and a summary of the free text comments. Similar to the first round, an opportunity to comment on the statements was offered in the form of a free text field. The same analytic approach was used concerning the possible consensus as in round 1.

### Data analysis

The answers were analyzed using Excel. Consensus was reached if > 75% of the panelists agreed (strongly agree/agree) or disagreed (strongly disagree/disagree) with the proposed care component. The cut-off of 75% was selected a priori.

## Results

### Panelists

The characteristics of the panelists are presented in Table 1. Ninety-two persons were invited to participate in the Delphi survey. In total, 63 panelists participated in the first round and 55 out of the 63 who participated in round one in the second round from 28 hospitals or long-term care facilities. The main professional background was geriatrics (35% in the first round, 36% in the second round) and trauma surgery/orthopaedics (22% in the first round, 20% in the second round). Most panelists worked in a non-academic hospital (67% and 65%). The median years of experience in hip-fracture care of the panelists were ten years in both rounds ([interquartile range (IQR)] = 5–15). In total, 45% (*N* = 25) of the panelists in the second round worked in an orthogeriatric unit with shared responsibilities between the surgical and geriatric departments.

**Table 1:** Sociodemographic details of Delphi expert panel

	First round n (%) total *n* = 63	Second round n (%) total *n* = 55
Respondent background		
Anaesthesiology	4 (6%)	3 (5%)
Emergency care	2 (3%)	2 (4%)
Elderly care medicine	5 (8%)	5 (9%)
Geriatrics/geriatric internists	22 (35%)	20 (36%)
Surgery/orthopaedic	14 (22%)	11 (20%)
Allied health professionals*	10 (16%)	8 (15%)
Patients/adults (with relatives who sustained a fracture)	6 (10%)	6 (11%)
Current work setting		
Academic hospital	5 (8%)	4 (7%)
Non-academic hospital	42 (67%)	36 (65%)
Ambulatory	10 (16%)	9 (16%)
Not applicable	6 (10%)	6 (11%)
Age (median, IQR)	42 [36–48]	43 [36–48]
Years of experience in hip fracture care (median, IQR)	10 [5–15]	10 [6–15]
Working in an orthogeriatric unit with shared responsibilities (yes)	Not collected	25 (45%)

*First round: 3 occupational therapists, 6 physiotherapists, 1 dietitian; Second round: 2 occupational therapists, 5 physiotherapists, 1 dietitian

### Delphi rounds

**Fig. 1:**
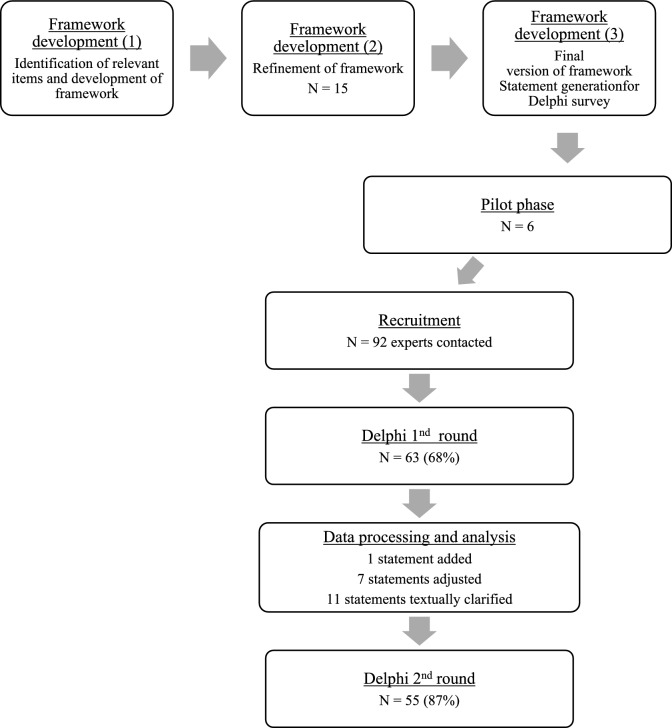
Number of panelists and response percentage in two Delphi rounds of the Delphi study including adjustments after the first round

**Fig. 2:**
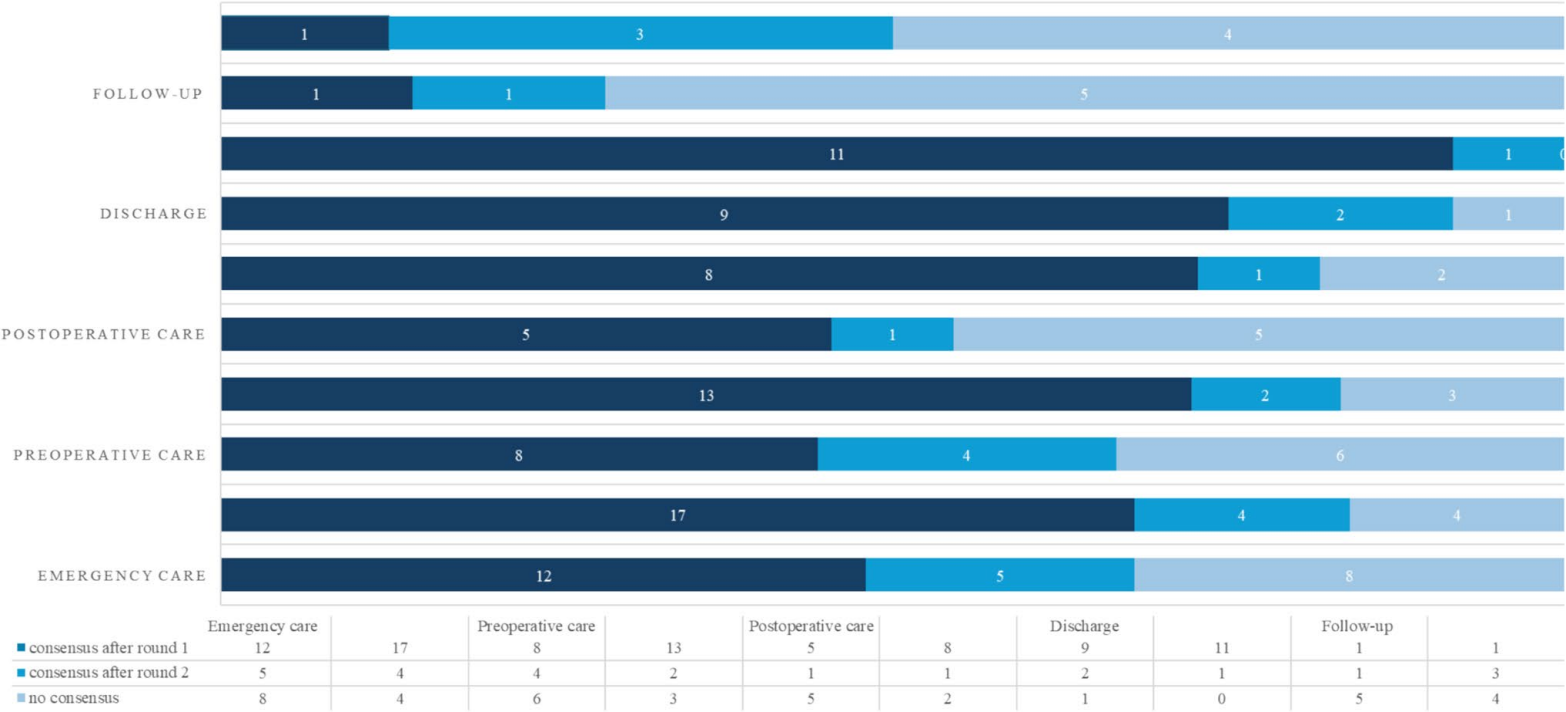
Number of statements that reached consensus after round 1 and 2 divided per stage of care

Details on all statements on which consensus was reached are presented in Table 2. In total, 72 statements were presented to all panelists in the first round. Thirty-five statements consensus was reached in round one for core elements of minimal orthogeriatric care, and 50 consensus was reached in round one for optimal orthogeriatric care (Fig. 1) After round one, one statement was added, seven statements were adjusted, and 11 statements were rephrased to increase comprehension. In round two, 38 statements were presented. After two rounds, out of the total 73 statements, 48 consensus was reached on minimal orthogeriatric care, and 60 consensus was reached on optimal orthogeriatric care (Fig. 2).

**Table 2:** All statements that have reached consensus (defined as > 75% agreement) for minimal and optimal care

Statements	Minimal care	Optimal care
*Emergency care*		
The components mentioned in the referral letter must align with local protocols and NHG guidelines	✓	✓
The treatment limitation policy must be specified in the referral letter. (this refers to any established policy ranging from full policy to entirely restricted policy)	✓	✓
If the SDM* does not provide a clear consensus or is medically complex, an ad-hoc MDT at the Emergency Department should be possible:		
With the involvement of the geriatrics/elderly care specialty through intercollegiate consultation (after physical assessment by this specialty)		✓
With the involvement of the general practitioner or elderly care physician		✓
If the SDM* does not yield a clear consensus or involves medical complexity, a follow-up discussion on the subsequent day is customary, with the patient being admitted specifically for this purpose	✓	✓
A hip fracture patient stays at most 90 min in the Emergency Department		✓
For every hip fracture patient, a fast track should be available		✓
At the Emergency Department, the following diagnostics should be performed (in addition to identifying the hip fracture on X-hip or CT):		
General blood test	✓	✓
Comprehensive blood test**	✓	✓
Chest x-ray***	✓	✓
ECG	✓	✓
Urine sediment		✓
Bladder scan	✓	✓
Regional anesthesia (e.g., femoral block) must be offered to every hip fracture patient if there are no contraindications and the surgery is not expected to occur within 6 h	✓	✓
Medication verification must occur within 24 h after presentation at the Emergency Department	✓	✓
The involvement of the geriatrics/internal elderly medicine specialty should take the form of		
Consultation	✓	
Comanagement	✓	✓
Involvement of the geriatrics/internal elderly medicine specialty in the form of physical assessment must take place:		
Preoperatively	✓	✓
Post-operatively	✓	✓
With every patient, a conversation focused on expectation management must take place, specifically regarding the expected course of admission, treatment and rehabilitation goals, and expectations	✓	✓
To each patient, information regarding the expected course of admission and treatment goals must be provided (e.g., in the form of an informational brochure)	✓	✓
A fall analysis, including a fall risk assessment, must be conducted during admission.**	✓	✓
*Shared decision making*		
For patients with a good performance score (ASA 1–3), an SDM should take place regarding the type of treatment, such as the (type of) implant or prosthesis	✓	✓
For patients with a good performance score (ASA 1–2), an SDM should take place regarding the type of anesthesia	✓	✓
For patients with a poor performance score (ASA 4–5, BMI < 18, or lack of independent mobility), an SDM should take place regarding whether or not to undergo surgical treatment	✓	✓
The conditions for an SDM are adequately documented in a manual or local protocol. This includes at least creating a calm environment, involving family members, and, if necessary, consulting or involving the patient’s general practitioner, geriatrician, or other medical specialist beforehand	✓	✓
*Nonoperative treatment*		
Adequate local pain management (including PENG block/phenolization) should be offered as standard or discussed as a treatment option for nonoperative treatment alongside systemic analgesics (tablets, patches, etc.)	✓	✓
The active involvement of family members should be an integral part of NOM	✓	✓
Adequate information and expectation management should be part of NOM, both verbally and in writing	✓	✓
Discharge to the patient’s living environment is aimed for, as long as palliative care can be provided there	✓	✓
*Preoperative Care*		
Each hip fracture patient must have been discussed at least once during an MDT meeting to optimize treatment and rehabilitation, including appropriate aftercare on the ward involving all relevant specialties		✓
The following allied health professionals must be consulted preoperatively:		
Physiotherapist	✓	✓
Dietician (in case of malnutrition)	✓	✓
Standard delirium scoring should be maintained	✓	✓
Standard delirium preventive measures should be implemented for every patient > 70 years old with a hip fracture	✓	✓
In the context of delirium prevention, every patient must be offered the opportunity for ‘rooming-in.’		✓
The local care pathway should be integrated into the EPR		✓
*Postoperative care*		
On day 0 (immediately postoperative), mobilization should be initiated if logistically feasible	✓	✓
The following allied health professionals must be consulted post-operatively:		
Physiotherapy	✓	✓
Dietitian		✓
The aftercare process must be initiated immediately post-operatively	✓	✓
If hip fracture care is provided in a hospital, a GTU must be available. (In a GTU, there is at least equal treatment authority between surgical and medical specialties, and care is provided based on geriatric principles)		✓
A GTU should have a living room where patients are offered daytime activities and eat together	✓	✓
A complete CGA should be conducted for every patient in which geriatrics/internal elderly medicine is involved		✓
A shortened CGA should be conducted for every patient whose geriatrics/internal elderly medicine is involved	✓	✓
A medication review should be conducted for every patient in which geriatrics/internal elderly medicine is consulted or involved in co-management	✓	✓
*Discharge*		
During admission, osteoporosis treatment (calcium, vitamin D, bone resorption inhibitor) should be initiated for patients over 75 years with hip fractures	✓	✓
There is a care pathway to follow-up on the care for underlying osteoporosis	✓	✓
Osteoporosis and the treatment are mentioned in the discharge letter	✓	✓
A monthly evaluation of complications that occurred in all hip fracture patients must take place		✓
A standard discharge discussion must take place focusing on expectation management in the presence of family members	✓	✓
There must be clear agreements regarding rehabilitation care documented in a local protocol	✓	✓
There must be a standard letter available that contains the following elements:		
Course of admission	✓	✓
Any complications	✓	✓
Weight-bearing policy	✓	✓
Interventions	✓	✓
Treatment limitation policy	✓	✓
Upon discharge, the patient should receive a letter that addresses, among other things, expectation management, capacity, and goals	✓	✓
*Follow-up care*		
A follow-up X-ray must be routinely performed after 6 weeks.**** (Disagreed)		✓
Patients should be seen at the outpatient clinic after 6 weeks, only if necessary	✓	✓
Based on indication, patients should receive an appointment for osteoporosis management at the outpatient clinic within 3 months aimed at diagnostics and treatment. (This applies to patients for whom no treatment has been initiated in the hospital)		✓
If there is an indication for long-term care after admission within geriatric rehabilitation care, whether follow-up appointments are still meaningful should be reassessed	✓	✓

*NHG* Het Nederlands huisartsgenootschap, *SDM* shared decision making, *MDT* multidisciplinary team, *CT* computer tomography, *ECG* electrocardiogram, *EPR* electronic patient record, *GTU* geriatric trauma unit, *CGA* comprehensive geriatric assessment

*Focused on operative versus nonoperative management, **According to the guideline Comprehensive Geriatric Assessment. ***On indication in case of abnormal physical examination or vital signs. ****for this statements panelists reached consensus on disagreement

### Consensus and qualitative feedback per stage of care

#### Emergency department

Consensus was reached regarding the role of geriatric care: minimal orthogeriatric care required consultation of a geriatrician, while optimal orthogeriatric care required co-management by the geriatric and surgical team. Regarding the timing of consultations, including physical assessments, experts reached a consensus on conducting them before or after surgery for minimal and optimal care. One panelist added, ‘ Physical assessment by a geriatrician is an admirable goal, but potentially not feasible for every patient.’ Concerning the statement: ‘A hip fracture patient stays a maximum of 90 min in the Emergency Department’, which reached consensus for optimal care, several panelists noted that ‘Attention for all aspects of the patient takes time and should not be rushed’ and ‘This may be possible for simple patients but not for all patients.’

#### Preoperative care

Panelists agreed on the consultation of the physiotherapist and dietician (in case of malnutrition) for minimal and optimal orthogeriatric care. The statement: ‘Assessing the patient’s ability to swallow by a speech pathologist’ reached no consensus (29% minimal and 56% optimal). Consultation by an occupational therapist did not reach a consensus for both minimal and optimal care (23% minimal and 46% optimal). Several panelists noted: ‘Consultation of the speech and occupational therapists should be available only on indication.’

### Shared decision making

Statements regarding SDM were posed for diverse patient categories and contexts. For patients with a good performance score, panelists agreed that SDM should occur based on the type of treatment, such as (type of) implant or prosthesis, and type of anesthesia for both minimal and optimal care. For patients with a poor performance score (ASA 4–5, BMI < 18, or lack of independent mobility), panelists agreed on SDM regarding whether or not to undergo surgical treatment for both minimal and optimal care.

### Nonoperative management

In the case of NOM regarding minimal and optimal care, panelists agreed on the statement, ‘Adequate local pain management (including pericapsular nerve group block (PENG) block/phenolization) should be offered as standard or discussed as a treatment option for nonoperative treatment alongside systemic analgesics (tablets, patches, etc.).’ Yet one panelist noted that ‘PENG block is not available in every hospital and cannot be administered by every anesthesiologist.’

#### Postoperative care

Panelists reached a consensus on the statement: ‘If hip fracture care is provided in a hospital, a geriatric trauma unit (GTU) must be available’ for optimal care (82%) but not for minimal care (63%). GTU was defined as “In a GTU, there is at least equal treatment authority between surgical and medical specialties, with care provided based on geriatric principles.” Concerning the design of the GTU, panelists reached a consensus on the statement: ‘A GTU should have a living room where patients are offered daytime activities and eat together.’ for minimal and optimal care (79% and 90%).

#### Discharge

Panelists reached a consensus on the management of osteoporosis. It was agreed that upon admission, patients aged over 75 with hip fractures should receive osteoporosis treatment, including calcium, vitamin D, and bone resorption inhibitors (78% for minimal and 91% for optimal). The following statement: ‘Upon discharge, the patient should receive a letter that addresses, among other things, expectation management, capacity, and goals.’ reached consensus for minimal (82%) and optimal (88%) care.

#### Outpatient care

Overall, there were a few statements on which consensus was reached concerning the outpatient care phase. Regarding minimal care, consensus was reached on two out of seven statements and for optimal care on four out of seven statements. For an appointment on indication at 6 weeks, consensus was reached regarding both minimal and optimal care. Several panelists noted regarding standard follow-up appointments: “Standard check-ups should be avoided. If there is no added value or specific request for help, these are an additional burden for the patient, their relatives, and the healthcare providers.” Consensus was reached on the following statement: ‘If a need for long-term care arises during admission to geriatric rehabilitation, it is important to reassess the necessity of follow-up appointments’ for minimal and optimal care (90% and 91%, respectively).

## Discussion

This study formulated the elements for minimal and optimal orthogeriatric care for hip-fracture patients using the Delphi method. Out of the total 73 statements, 48 were reached for minimal orthogeriatric care; 60 consensus was reached for optimal orthogeriatric care, highlighting a gap of 12 statements between the minimal and optimal standards of care. Several key elements were solely identified for optimal orthogeriatric care but not minimal orthogeriatric care. For example, the availability of a geriatric trauma unit if a hospital provides hip-fracture care and evaluation of every patient during a multidisciplinary team (MDT) meeting. In addition, recommendations were identified on novel domains e.g., regarding the care for patients treated non-operatively.

The results of this Delphi survey are largely in line with current guidelines and the advice given by the FFN and underscore one of the pillars of the FFN: ‘Multidisciplinary care of the acute fracture episode along orthogeriatric lines.’ [6]. Some results, however, are contradictive to current guidelines or to the FFN advice and therefore further discussed.

Consistent with the FFN recommendations, panelists agreed that osteoporosis treatment should begin during hospital admission, and there should be a care pathway specifically for this management. A recent study by Johansen (2023) pleaded for protocols to provide osteoporosis treatment with intravenous zoledronate as the standard of care aligns with this statement. [19] This aligns with the recently updated Dutch guideline for fracture prevention and osteoporosis. The guideline advises administering zoledronic acid for hip-fracture patients aged 75 years and older within three months. In this study, consensus was only reached regarding a follow-up appointment for osteoporosis management for optimal but not minimal orthogeriatric care. Several panelists reasoned that out-of-hospital healthcare professionals may also provide this type of treatment; therefore, the follow-up should not necessarily occur in the outpatient clinic. This is a deviation from the current Dutch guideline, which advises evaluating every patient three months after initial treatment. [11]

Concerning NOM, the panelists reached a consensus that for patients with a poor performance score, a SDM should be used to determine whether to undergo surgical treatment. This is partly in line with the current Dutch guideline from 2016, which states that for ASA 3–5, a nonoperative treatment may be considered after an SDM if there is an explicit wish [11]. Recent research showed that for frail patients living in a nursing home with extensive functional disabilities, NOM resulted in similar outcomes regarding quality of life when compared to operative management for this small group of frail patients [20]. However, The FFN states that conservative treatment should be avoided in modern healthcare systems, except for terminally ill patients. Previous research also showed heterogeneity in the factors influencing clinicians' decisions to opt for NOM [21]. In addition, variations between countries may exist, and this statement is assessed specifically within the Dutch context. Recent developments and emerging evidence regarding NOM and ongoing debates suggest that current guidelines and the FFN may not fully reflect real-world practice. This indicates a gap between established protocols and practical implementation. Given the complexity of this type of care, further elaboration on this topic is needed to inform clinicians and patients and provide guidelines.

In this study, consensus was reached for the joint care between surgical care and geriatricians regarding either co-management for optimal orthogeriatric care or co-management or consultation for minimal orthogeriatric care. A recent study by Werner et al. revealed that the Netherlands scored the lowest among all European countries with a percentage of orthogeriatric co-management at 74% [22]. In addition, an evaluation of care pathways in the Netherlands showed an equal distribution of integrated care models and surgeon-led care [23]. Therefore, there appears to be a gap between practice and the outcome of recommendations formed in this study and the current Dutch guidelines and evidence in the literature that recommends co-treatment by a geriatrician [12]. The background of the panelists may also reflect the gap since 45% worked in an orthogeriatric unit with shared responsibilities. This may have led to a consensus for a relatively higher standard of care for the minimal care components and a smaller difference between minimal and optimal care.

A notable number of respondents commented on different statements that in the ‘ideal’ situation, the statement should be satisfied. Still, implementation requires major logistical changes (e.g., room for rooming in) or organizational changes (e.g., a fall analysis must be conducted during admission) in the hospitals. This is in line with the study of Gupta et al., which showed an understanding of the benefit of a geriatrics-surgery co-management program but also identified several facilitators for implementation, such as the availability of geriatrician and administration support [24]. Thus panelists still acknowledged the disparity between the ideal scenario and the current practices regarding efficiency versus patient-centered care and the 'ideal' scenario and 'local' challenges. Furthermore, some formulated recommendations on core and optimal elements conflict with the evidence presented in the literature. For example, no consensus was reached on whether to assess the ability to swallow by a speech pathologist. However, there are arguments supporting its implementation, especially given the high incidence of aspiration pneumonia among patients, which is associated with high mortality [25, 26]. This may show the lack of awareness of this topic’s current literature, which could be resolved by more research and education.

The limitation of this study is the extensive number of statements (73 statements), which may have influenced the response percentage [27]. Second, the CREDES was originally developed to develop and report Delphi studies in palliative care. However, as stated in the discussion of the CREDES report, CREDES can be applied outside of palliative care [17]. In addition, care for hip-fracture patients is extensive and involves many care phases of the healthcare system; therefore, not every aspect could be assessed in this study. The generalizability of the results in this study should be viewed in the context of the historical, cultural, organizational, and financial conditions of the Dutch healthcare system, which may limit the broader applicability of the findings. The strength of the study lies in the multidisciplinary nature of the panelists. In addition, six patient representatives were included in the study, as the researchers acknowledged the importance of incorporating the patient perspective within the research. Another strength is the practical applicability of these elements in diverse clinical settings and stages of development.

## Conclusion

The outcomes of this study provide guidance and gives both recommendations for minimal and optimal care, promoting self-assessment against the benchmarks and implementation of recommendations. The recommendations in majority align with the advice given on orthogeriatric care for older adults with hip fractures as outlined by the FFN and clinical guidelines. In addition, recommendations were formed for NOM and shared decision-making. Moving forward, this research may decrease the variability in the clinical management of hip-fracture patients. However, several organizational and logistical barriers need to be addressed to bridge the gap between the ideal situation and the current practices.

## Data Availability

The data used in this study are available upon request. Interested researchers may contact the corresponding author. Access to the data will be provided after approval of the authors. To maintain confidentiality, no traceable data will be shared. The data provided will be on an aggregated level, not at the level of individual panelists.
